# Prevalence and Characteristics of Ceftriaxone-Resistant *Salmonella* in Children’s Hospital in Hangzhou, China

**DOI:** 10.3389/fmicb.2021.764787

**Published:** 2021-11-22

**Authors:** Qiucheng Shi, Yihua Ye, Peng Lan, Xinhong Han, Jingjing Quan, Mingming Zhou, Yunsong Yu, Yan Jiang

**Affiliations:** ^1^Department of Clinical Laboratory, The Children’s Hospital, Zhejiang University School of Medicine, National Clinical Research Center for Child Health, National Children's Regional Medical Center, Hangzhou, China; ^2^Department of Critical Care Medicine, Sir Run Run Shaw Hospital, Zhejiang University School of Medicine, Hangzhou, China; ^3^Department of Infectious Diseases, Sir Run Run Shaw Hospital, Zhejiang University School of Medicine, Hangzhou, China; ^4^Key Laboratory of Microbial Technology and Bioinformatics of Zhejiang Province, Hangzhou, China; ^5^Regional Medical Center for National Institute of Respiratory Diseases, Sir Run Run Shaw Hospital, Zhejiang University School of Medicine, Hangzhou, China

**Keywords:** ceftriaxone resistance, children, China, extended-spectrum beta-lactamase, non-Typhi *Salmonella*

## Abstract

The non-Typhi *Salmonella* (NTS) infection is critical to children’s health, and the ceftriaxone is the important empirical treatment choice. With the increase resistance rate of ceftriaxone in *Salmonella*, the molecular epidemiology and resistance mechanism of ceftriaxone-resistant *Salmonella* needs to be studied. From July 2019 to July 2020, a total of 205 NTS isolates were collected, 195 of which (95.1%) were cultured from stool, but 10 isolates were isolated from an extraintestinal site. Serogroup B accounted for the vast majority (137/205) among the isolates. Fifty-three isolates were resistant to ceftriaxone, and 50 were isolated from children younger than 4years of age. The resistance rates for ceftriaxone, ciprofloxacin, and levofloxacin were significantly higher in younger children than the older children. The resistance genes in the ceftriaxone-susceptible isolates were detected by PCR, and ceftriaxone-resistant *Salmonella* were selected for further whole-genome sequencing. Whole-genome analysis showed that serotype Typhimurium and its monophasic variant was the most prevalent in ceftriaxone-resistant isolates (37/53), which comprised ST34 (33/53), ST19 (2/53), and ST99 (2/53), and they were close related in the phylogenetic tree. However, the other isolates were diverse, which included one Enteritidis (ST11), one Indiana (ST17), one Derby (ST40), four Kentucky (ST198), two Goldcoast (ST2529, ST358), one Muenster (ST321), one Virchow (ST359), one Rissen (ST469), one Kedougou (ST1543), two Uganda (ST684), and one Kottbus (ST8839). Moreover, CTX-M-55 ESBLs production (33/53) was found to be mainly responsible for ceftriaxone resistance, followed by *bla*_CTX-M-65_ (12/53), *bla*_CTX-M-14_ (4/53), *bla*_CTX-M-9_ (2/53), *bla*_CTX-M-64_ (1/53), *bla*_CTX-M-130_ (1/53), and *bla*_CMY-2_ (1/53). IS*Ecp1*, IS*903B*, IS *Kpn26*, IS*1F*, and IS*26* were connected to antimicrobial resistance genes transfer. In conclusion, the dissemination of ESBL-producing *Salmonella* isolates resulted in an increased prevalence of ceftriaxone resistance in young children. The high rate of multidrug resistance should be given additional attention.

## Introduction

*Salmonella* is a genus of Gram-negative bacteria of the family Enterobacteriaceae, and *Salmonella enterica subsp. enterica* is the most frequent pathogen triggering human sickness (Smith et al., 2016). *Salmonella* infection is officially called as salmonellosis, which causes stomach cramps, diarrhea, and fever. Salmonellosis is usually related to contaminated foods, including raw meat and fruits, unpasteurized milk and dairy products, and undercooked eggs (Wegener et al., 2003). Salmonellosis is a global health issue, with approximately 93 million cases of gastroenteritis and 155,000 associated deaths caused by *Salmonella* every year worldwide (Majowicz et al., 2010).

Serodiagnosis signatures were first recognized for classification in medical routine practice, and *Salmonella* spp. can be divided into *Salmonella* Typhi, *Salmonella* Paratyphi (A, B, or C), and non-Typhi *Salmonella* (NTS; Switt et al., 2009). In contrast to *Salmonella* Typhi and *Salmonella* Paratyphi that are rarely encountered outside endemic countries or in returning travelers, NTS has a high worldwide distribution. NTS infections are usually self-limiting, and antibiotics are now not indicated for uncomplicated infections. However, antibiotic therapy needs to be considered for populations at increased risk for invasive infections, such as neonates and elderly person, and antimicrobial treatment should also be modified or discontinued when a clinically plausible organism is identified (Shane et al., 2017).

Ceftriaxone is a third-generation cephalosporin with excellent activity against many Gram-negative microorganisms (Nahata and Barson, 1985) and is the empirical choice for *Salmonella* spp. treatment. With the misuse and overuse of antibiotics, as well as poor infection prevention and control, antibiotic resistance has accelerated over time, which threatens public health and clinical treatment. The most important resistance mechanism of ceftriaxone is producing AmpC β-lactamases, such as CMY and DHA, and extended spectrum β-lactamases (ESBLs), which include the TEM, SHV, CTX-M, VEB, and GES enzymes (Nahata and Barson, 1985).

In this study, we aimed to evaluate the molecular epidemiology of *Salmonella* spp. in children and to reveal the resistance mechanism and transmission pattern of ceftriaxone-resistant *Salmonella*. For the purpose, we collected the *Salmonella* spp. from Children’s Hospital from July 2019 to July 2020, and antimicrobial susceptibility tests and seroagglutination tests were conducted for these isolates. After that, we analyzed the genome characteristics and phylogenetic relationship of ceftriaxone-resistant *Salmonella* spp. by whole-genome sequencing, moreover, analyzed the genetic background mediated the ceftriaxone resistance.

## Materials and Methods

### Collection of Isolates and Clinical Information

In Children’s Hospital of Zhejiang University School of Medicine from July 2019 to July 2020, all patients for whom clinical specimen were collected and found to host *Salmonella* were included in this study. For the routine work in the department of clinical laboratory, briefly, stool samples were inoculated onto blood agar (BIOIVD, Zhengzhou, China) and SS agar (Comagal, Shanghai, China) and cultured at 35°C for 18h. Blood samples were cultured with a Bactec FX400 System (BD, NJ, United States), and the other types of samples were incubated on blood agar (BIOIVD, Zhengzhou, China). Then, the correct clones were picked and streaked to purity for the tests. Patients’ information, including age, sex, and infection site, was obtained from the digital record system, and their names were masked. The age of children was calculated in decimal years by considering a month approximately 0.083years, and a day approximately 0.0027years. This study was approved by the Ethics Committee of Children’s Hospital of Zhejiang University School of Medicine (2021-IRB-031).

### Bacterial Isolate Identification and *Salmonella* Serotyping

All the isolates were identified by matrix-assisted laser desorption ionization-time of flight (MALDI-TOF) mass spectrometry systems (Bruker Daltonics, Bremen, Germany). The serogrouping was conducted by a seroagglutination test (Tianrun, Ningbo, China), and serogroups A, B, C1, C2-C3, D1, and E1 were determined. These serogroups were also identified as O:2, O:4, O:7, O:8, O:9, and O:3, 10, respectively.

### Antimicrobial Susceptibility Testing

Antimicrobial agent susceptibility was tested for all collected isolates, including against ampicillin, ceftriaxone, chloramphenicol, ciprofloxacin, levofloxacin, meropenem, and trimethoprim/sulfamethoxazole. *Escherichia coli* ATCC 25922 was used as the quality control. The agar dilution method was used following the guidelines of the Clinical & Laboratory Standards Institute (CLSI), and the breakpoint was interpreted according to the CLSI guidelines (M100 29th edition). The AST of colistin was determined by the micro broth dilution method, and a minimal inhibitory concentration (MIC)>2mg/L was considered resistant following European Committee on Antimicrobial Susceptibility Testing (AST; EUCAST) breakpoint interpretation.

### Whole Genome Sequencing

Ceftriaxone-resistant *Salmonella* were selected for whole-genome sequencing, and genomic DNA was extracted with a QIAamp DNA Mini Kit (QIAGEN, Hilden, Germany). The Illumina HiSeq X-Ten platform (Illumina, San Diego, CA, United States) was chosen for whole-genome sequencing with a 150bp paired-end strategy, as previously described (Shi et al., 2020). Then, the reads were assembled by Shovill,^1^ and unqualified contigs (coverage <10 or length<200bp) were eliminated from subsequent analysis.

### Detection of the Multilocus Sequence Typing, Serovar, and Antimicrobial Resistance Genes

Detection of the multilocus sequence typing (MLST) and the Inc-type plasmid of the strain were screened and determined by using the MLST 2.0 server and Plasmid Finder 1.3 at the Center for Genomic Epidemiology,^2^ respectively (Larsen et al., 2012; Carattoli et al., 2014), and the molecular serovar was identified by SISTR (Yoshida et al., 2016) on Pathogenwatch.^3^ Monophasic *Salmonella* Typhimurium (1, 4, [5], 12: i: -) was classified as *Salmonella* Typhimurium (1, 4, [5], 12: i: 2).

For ceftriaxone-resistant isolates, antimicrobial resistance genes (ARGs) were identified with whole genome sequences (WGSs) by ABRicate with parameter identity >90% and minimum length>60%,^4^ and the National Center for Biotechnology Information (NCBI) bacterial antimicrobial resistance reference gene database was set as the reference (Feldgarden et al., 2019). The *bla*_TEM_, *bla*_CTX-M-1_ group, *bla*_CTX-M-9_ group, and *bla*_CMY-2_ among the ceftriaxone susceptible isolates were screened by polymerase chain reaction (PCR) and Sanger sequencing, and the primers used in this study are shown in Supplementary Table S1.

### The Genetic Structure Surrounding of ESBLs

The contigs that contained ESBL genes were extracted and annotated by Prokka (Seemann, 2014), and particularly by ISfinder for identifying mobile genetic elements (MGEs; Siguier et al., 2006).^5^ The transposons or translocatable units that were responsible for ESBL gene transfer were ascertained and their genetic structure was visualized.

### Phylogenetic Relationship Analysis

All 53 ceftriaxone-resistant isolates were imported for phylogenetic relationship analysis based on their single-nucleotide polymorphisms (SNPs). The genome alignment was established by Snippy with default parameters,^6^ and one internal strain, SM-1 (accession number: SAMN20422865), was used as a reference, and the SNPs numbers between each isolate were calculated by Snp-dists.^7^ Fasttree (version 2.1.11) and generalized time-reversible model was used in phylogeny calculation, and the tree was illustrated and annotated by Evolview (Subramanian et al., 2019).

### The Statistical Analysis

The comparison of antimicrobial resistance rates was performed by Fisher’s exact, and *p*<0.05 was considered as statistically significant.

### Nucleotide Sequence Accession Numbers

The WGS of ceftriaxone-resistant *Salmonella* spp. were deposited in the NCBI database under BioProject accession number PRJNA749852.

## Results

### Patients and Strains

During the period of the current study, a total of nonduplicated 205 *Salmonella* isolates were obtained. The isolates from feces accounted for the vast majority (195/205), and the remaining 10 isolates were from several types of sterile body fluids. Six out of 10 were from blood samples in the gastroenterology department, respiratory department, neonatal department, and pediatric intensive care unit (PICU). One strain (SM-201) was isolated from pleural fluid and the other (SM-290) was isolated from abdominal pus (general surgery department). There were two isolates from subperiosteal pus (SM-103 and SM-301, orthopedics department). A total of 86.8% (178/205) of *Salmonella* isolates were from children less than 4years of age in this study, and the median age of these children was 1.25 (IQR: 0.79 to 2.29), suggesting that younger children were the most potentially susceptible population.

### Antimicrobial Susceptibility and Serotyping of *Salmonella* spp.

The seroagglutination test and AST were performed to explore the distribution of *Salmonella* serogroups and antimicrobial susceptibility in a tertiary children’s hospital (Table 1). Fifty-three ceftriaxone-resistant isolates were found in these collected *Salmonella* isolates and resistance rate reached 25.9%, and the other antimicrobial resistance rates for chloramphenicol, ciprofloxacin, levofloxacin, ampicillin, and trimethoprim/sulfamethoxazole were 44.4% (91/205), 23.4% (48/205), 11.7% (24/205), 75.6% (155/205), and 37.1% (76/205), respectively. The multidrug resistance (MDR) rate in the current study was 37.6% (77/205), and there was no meropenem-resistant *Salmonella*. Interestingly, the resistance rates for ceftriaxone, ciprofloxacin, and levofloxacin were significantly higher in younger children (age<1) (Supplementary Figure S1), and 94.3% (50/53) of ceftriaxone-resistant *Salmonella* isolates were distributed in children less than 4years of age. Furthermore, the resistance rates for ciprofloxacin and levofloxacin in the ceftriaxone-resistant group were significantly higher than those in the ceftriaxone-susceptible group (*p*=0.0006 and<0.0001, respectively). For instance, the ciprofloxacin resistance rate was 41.5% (22/53) in the ceftriaxone-resistant group, compared with 17.1% (26/152) in the ceftriaxone-susceptible group; and the difference resistance rates were 34.0% (18/53, ceftriaxone resistant) vs. 3.9% (6/152, ceftriaxone susceptible) for levofloxacin.

**Table 1:** The clinical information, serogroup and antibiotic resistance rate of 205 *Salmonella* isolates.

	Age (<1)	Age (1–4)	Age (≥4)	Total
**Isolates number**	81	97	27	205
**Age (medium, IQR)**	0.75 (0.46, 0.9)	1.60 (1.25, 2.29)	5.58 (4.91, 8)	1.25 (0.79, 2.29)
**Gender (male)**	50.6 (41/81)	55.7 (54/97)	59.3 (16/27)	53.9 (111/205)
**Resistance rate**				
Ceftriaxone	33.3 (27/81)	23.7 (23/97)	11.1 (3/27)	25.9 (53/205)
Chloramphenicol	50.6 (41/81)	43.3 (41/97)	33.3 (9/27)	44.4 (91/205)
Ciprofloxacin	33.3 (27/81)	17.5 (17/97)	14.8 (4/27)	23.4 (48/205)
Levofloxacin	21.0 (17/81)	5.2 (5/97)	7.4 (2/27)	11.7 (24/205)
Meropenem	0 (0/81)	0 (0/97)	0 (0/27)	0 (0/205)
Ampicillin	77.8 (63/81)	71.1 (69/97)	85.2 (23/27)	75.6 (155/205)
SMZ	43.2 (35/81)	34.0 (33/97)	29.6 (8/27)	37.1 (76/205)
**Serogroup**				
B	64.2 (52/81)	76.3 (74/97)	40.7 (11/27)	66.8 (137/205)
C1	6.2 (5/81)	10.3 (10/97)	14.8 (4/27)	9.3 (19/205)
C2-C3	9.9 (8/81)	1.0 (1/97)	3.7 (1/27)	4.9 (10/205)
D	4.9 (4/81)	8.2 (8/97)	33.3 (9/27)	10.2 (21/205)
E1	8.6 (7/81)	3.1 (3/97)	7.4 (2/27)	5.9 (12/205)
Unindentified	6.2 (5/81)	1.0 (1/97)	0 (0/27)	2.9 (6/205)

Serogroup determination by seroagglutination testing showed that serogroup B was the most prevalent type (137/205) among these *Salmonella* isolates, followed by serogroup D1 (21/205), serogroup C1 (19/205), serogroup E1 (12/205), and serogroup C2-C3 (10/205). There were six isolates that could not be classified into a specific serogroup by the seroagglutination testing. One of these isolates, SM-233, was resistant to ceftriaxone, and further whole genome sequencing showed that SM-233 belonged to serovar Kedougou, which is serogroup G *Salmonella* (Rodriguez-Urrego et al., 2010). Serogroup B *Salmonella* isolates also showed the most prevalent ceftriaxone resistance.

### The Molecular Characteristics of Ceftriaxone-Resistant *Salmonella* Isolates

Whole-genome sequencing data of ceftriaxone-resistant *Salmonella* isolates were utilized to further clarify their molecular characteristics and phylogenetic relationship. All the ceftriaxone-resistant *Salmonella* isolates were from stools except one from blood. The MLST results showed that ST34 was the predominant sequence type (33/53, 62.3%), and the others were diverse, including one ST11, one ST17, two ST19, one ST40, two ST99, four ST198, one ST2529, one ST321, one ST358, one ST359, one ST469, one ST1543, and two ST684. Furthermore, one novel ST (ST8839) was found with the MLST profile (*aroC* 844, *dnaN* 71, *hemD* 43, *hisD* 12, *purE* 190, *sucA* 20, *thrA* 18), and it was nearest to ST808, with the point mutation C102T in *aroC*.

Among these isolates, serovar Typhimurium and its monophasic variant were predominant, and 37 serovar Typhimurium and its monophasic variant isolates corresponded to more than one sequence type, which included ST19, ST34 and ST99. ST358 and ST2529 belonged to serovar Goldcoast. In the other serovars, there was a one-to-one relationship between the serovar and the MLST, for instance, serovar Kentucky corresponded to ST198, and serovar Uganda corresponded to ST684.

**Figure 1:**
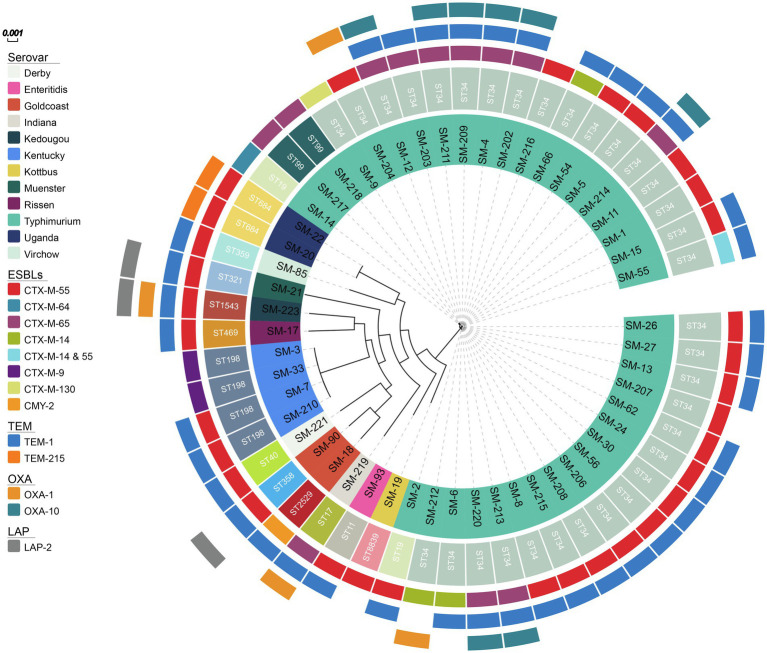
The phylogenetic tree of 53 ceftriaxone-resistant *Salmonella* from the inside to the outside, each circle represents the ST type, serovar, ESBL genes, *bla*_TEM_, *bla*_OXA_, *bla*_LAP_, respectively.

The phylogenetic tree showed that serovars, such as Enteritidis, Muenster, Rissen, Virchow, Indiana, Kedougou, and Kottbus, were differently positioned on the phylogenetic tree and unrelated (Figure 1). The results showed that the phylogenetic relationship among the same ST isolates was indistinguishable, such as among the ST684 serovar Uganda or among the ST198 serovar Kentucky. The mean number of SNPs between each pair of ST34 isolates was 52.5±22.7, indicating that the relationships within the predominant ST strains were relatively close. There were 23 indistinguishable pairs of isolates in ST34 that has less than 5 SNPs. SM-90 (ST358) isolated from stool and SM-18 (ST2529) isolated from blood were nearly unrelated, although both of them belonged to the serovar Goldcoast.

### Resistance Mechanisms of Ceftriaxone-Resistant *Salmonella* Isolates

**Figure 2:**
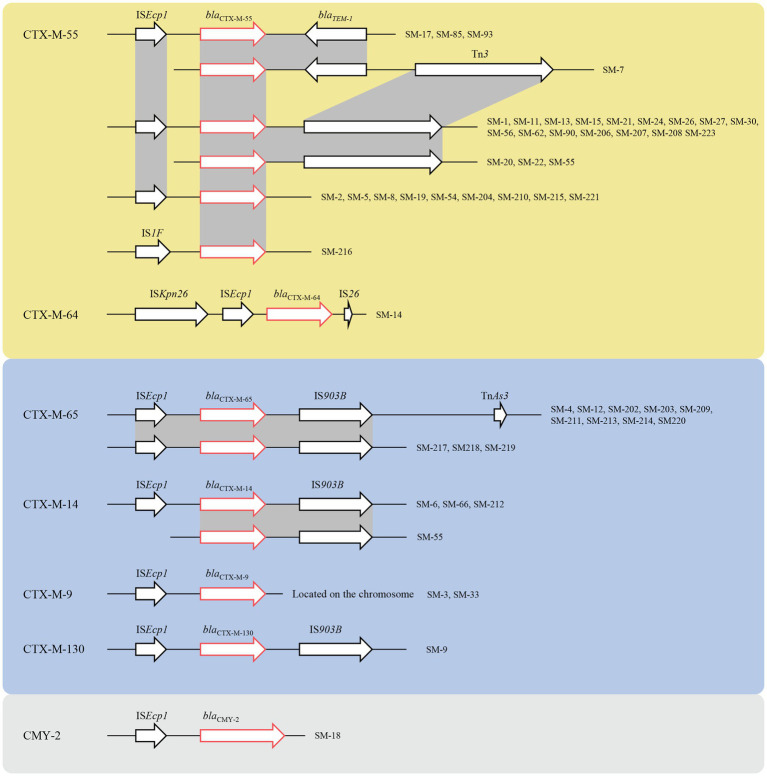
The genetic environment of ESBL, the yellow region represented for CTX-M-1 group, the blue region represented for the CTX-M-9 group, and the grey region represented CMY-2. The red arrows represented for the resistance genes, and the black arrows represented for the mobile genetic elements (MGEs).

To understand the ceftriaxone resistance mechanisms of these *Salmonella* spp. isolates, the antimicrobial resistance genes were analyzed. Almost all ceftriaxone resistant isolates were CTX-M-type ESBL producers, except one producer of CMY-2 β-lactamase, which is an AmpC cephalosporinase. In contrast, no CTX-M ESBLs or AmpC cephalosporinase genes were detected among the ceftriaxone-susceptible isolates, suggesting that those enzymes were possibly responsible for ceftriaxone resistance. Among the CTX-M-producing isolates, *bla*_CTX-M-55_ (33/53) was the most prevalent, followed by *bla*_CTX-M-65_ (12/53), *bla*_CTX-M-14_ (4/53), *bla*_CTX-M-9_ (2/53), *bla*_CTX-M-64_ (1/53), and *bla*_CTX-M-130_ (1/53). In particular, SM-55 harbored two different types of ESBLs, *bla*_CTX-M-14_ and *bla*_CTX-M-55_. TEM β-lactamases were mainly distributed (40/53) in ceftriaxone resistant isolates; however, the *bla*_TEM-1_ gene was still detected in 62.5% (95/152) of ceftriaxone susceptible isolates, showing its limited effect on ceftriaxone resistance. Among the ceftriaxone-resistant isolates, 38 isolates harbored *bla*_TEM-1_, and two isolates (SM-20 and SM-22) had *bla*_TEM-215_, which were both ST684. Four isolates were *bla*_OXA-1_ positive; eight isolates were *bla*_OXA-10_ positive, and there were three isolates contained *bla*_LAP-2_ (Figure 2).

Genetic environment analyses showed that the IS*Ecp1*-ESBL-IS*903B* structure was the most prevalent ESBL gene transfer. The CTX-M-1 group included CTX-M-55 and CTX-M-64, and the former had three different environments: IS*Ecp1*-*bla*_CTX-M-55_-IS*903B*, IS*Ecp1*-*bla*_CTX-M-55_-*bla*_TEM-1_-IS*903B*, and IS*1F*-*bla*_CTX-M-55_. Similarly, the gene structure of the latter was IS*Kpn26*-IS*Ecp1*-*bla*_CTX-M-64_-IS*26*. The CTX-M-9 group contained CTX-M-9, CTX-M-14, CTX-M-65, and CTX-M-130. The genetic structures of *bla*_CTX-M-14_, *bla*_CTX-M-65_, and *bla*_CTX-M-130_ were similar, which were mediated by IS*Ecp1* and IS*903B*, but *bla*_CTX-M-9_ was located on the chromosome, which was still mediated by IS*Ecp1*. The gene upstream of CMY-2 was IS*Ecp1*, which was located on the IncI1-I (Alpha) type plasmid (Figure 2).

### The Prevalence of *mcr*-1 in Ceftriaxone Resistant *Salmonella*

Interestingly, SM-66 and SM-212 both harbored the *mcr*-1 gene, which mediates resistance against colistin, the last-resort therapy choice for multidrug-resistant Gram-negative bacterial infection. The colistin MIC of isolate SM-212 (accession number: SAMN20422907) reached 8mg/L; however, the colistin MIC of isolate SM-66 (accession number: SAMN20422894) was just 0.5mg/L, remaining susceptible. WGS analysis showed that the deletion mutation (GTGGCGAGTGTTG) of *mcr*-1 in SM-66 was observed between nucleic acid positions 55 and 67. The plasmid type of these two *mcr*-1-harboring isolates was IncHI2, which is common plasmid groups carrying multidrug resistance determinants.

## Discussion

Gastroenteritis induced by NTS is usually self-limited, and antibiotics are usually not indicated for uncomplicated infection, as expected for children and immunocompromised patients (Pragasam et al., 2019). In the current study, in addition to gastrointestinal *Salmonella* infection, we found other invasive NTS (iNTS) infections, such as bloodstream infections, abdominal infection, and osteomyelitis, and even ceftriaxone-resistant *Salmonella* bloodstream infection, which caused an increased burden on clinical treatment. Chen’s report indicated that iNTS would cause prolonged hospitalization, increased medical costs, and elevated mortality (Chen et al., 2012).

The recommendations for the treatment of acute gastroenteritis due to NTS vary (Haeusler and Curtis, 2013), and the empirical choice of treatment is usually ceftriaxone or ciprofloxacin, but ciprofloxacin and other fluoroquinolones have been restricted in children (Stahlmann and Lode, 2013). Because of the clear side effects, which make ceftriaxone very important in NTS infection treatment for children. However, ceftriaxone-resistant *Salmonella* incidence has been increasing in recent year, and research has shown that the resistance rates to ceftriaxone in 2019 were significantly higher than those in 2012 (14.3% vs. 4.1%, *p*<0.001) (Chang et al., 2021), which presented increased challenges to clinical treatment. The results of Chinet, an annual national surveillance in China, showed that the rate of resistance to ceftriaxone in 453 *Salmonella enterica* isolates was only 5.7% in 2020, and the rate of resistance to ceftriaxone in 498 *Salmonella* Typhimurium isolates was 25.2%.^8^ In this study, the ceftriaxone resistance incidence was 25.9%. Previous research indicated that NTS isolates from children aged <5years showed a higher prevalence of antimicrobial resistance than those from patients >5years, with a similar elevated resistance rate to cephalosporins and quinolones (Liang et al., 2015), which is consistent with our results.

Ceftriaxone and ciprofloxacin had a tendency of co-resistance in this study; 40.7% (22/54) of ceftriaxone-resistant isolates also showed resistance to ciprofloxacin, and the percentage was higher than that in the ceftriaxone-susceptible group. The antimicrobial resistance genes ciprofloxacin and ceftriaxone are usually carried by plasmids, which facilitates the coexistence of these genes on the same plasmids. The results showed that strain SM-15 contained the *bla*_CTX-M-55_ and *qnrS1* on the same contig, which indicated that these genes were located on the same plasmid. Paterson indicated that ESBL gene harboring plasmids likely carry genes for resistance to many other antimicrobial agents, such as aminoglycosides, trimethoprim, sulfonamides, tetracyclines, and chloramphenicol, which may be the reason for MDR (Paterson, 2000). In these ceftriaxone-resistant *Salmonella* isolates, we found two *mcr*-1-positive isolates; interestingly, one of them was colistin susceptible, with a truncated *mcr*-1 allele. *mcr*-1-positive *Salmonella* isolates have been previously reported in Brazil (Rau et al., 2020), Spain (Trujillo-Soto et al., 2019), South Korea (Moon et al., 2021), and China (Lu et al., 2019), and the *mcr*-1 gene was found to be normally located on IncI2, IncHI2, and IncX4 plasmid types, respectively.

The serogroup of *Salmonella* is diverse worldwide. The majority serogroup of *Salmonella* in South India was serogroup B followed by E and C1/C2 (Pragasam et al., 2019), and the most prevalent ceftriaxone-resistant *Salmonella* serotype was serovar Heidelberg, followed by Newport in the United States (Medalla et al., 2016). In this study, we found that *Salmonella* Typhimurium represented the overwhelming majority of ceftriaxone-resistant *Salmonella*.

According to previous research, CMY-2 AmpC β-lactamase was the most important ceftriaxone resistance gene in *Salmonella* spp., followed by *bla*_CTX-M-3_ (Su et al., 2005). However, in our study, only one isolate contained CMY-2, and no isolates produced CTX-M-3. *bla*_CTX-M-55_ was the most common ESBL in this study, similar to a previous study in Shanghai (Li et al., 2021). The genetic structure of the ESBL gene was identical to that in a previous study. CTX-M and CMY-2 were in both the IS*Ecp1* and IS*903B* elements (He et al., 2021; Nguyen et al., 2021), and one CTX-M-64 was located in the IS*Kpn26*- IS*Ecp1*-*bla*_CTX-M-64_-IS*26* structure. In this study, *bla*_CTX-M-9_ was located on the chromosome, which was mediated by IS*Ecp1*, and became fixed. Maria et al. reported that the *bla*_CTX-M-55/57_ gene can transfer to *E. coli*, while repeated attempts to transfer plasmids harboring the *bla*_CTX-M-5_ and *bla*_CTX-M-15_ genes failed (Sjolund-Karlsson et al., 2011), which makes the prevalence of *bla*_CTX-M-55_ in our study explicable. Because ESBL genes are often carried by large plasmids (Rawat and Nair, 2010), we speculated that the plasmids carrying ESBL genes were transformed from other bacteria in the human intestinal tract, and further studies should be conducted to determine the origin and relationship of these resistance plasmids.

## Conclusion

In this study, we investigated the epidemiology of *Salmonella* in the children’s hospital in Hangzhou, China, which could supplement important local epidemiological data. Younger children were the potentially susceptible population for *Salmonella* infections, and ST34 *Salmonella* Typhimurium dominated the ceftriaxone-resistant strains. The major resistance mechanism of ceftriaxone-resistant *Salmonella* was producing CTX-M-type ESBLs, in which *bla*_CTX-M-55_ was the most prevalent. The dissemination of these ESBLs was mediated by mobile elements, including IS*Ecp1* and IS*903B*. In the current study, we found the co-transfer tendency of cephalosporin- and quinolone-resistance, and the increased prevalence of ceftriaxone resistance and the high-rate multidrug resistance should be concerned.

## Data Availability Statement

The datasets presented in this study can be found in online repositories. The names of the repository/repositories and accession number(s) can be found at: https://www.ncbi.nlm.nih.gov/, PRJNA749852.

## Author Contributions

YJ and YY participated in the design of the study. QS, YY, and MZ collected the isolates and clinical information. PL and YY performed the antimicrobial susceptible testing and serotyping. QS and XH prepared for whole-genome sequencing and PCR. JQ and XH performed the *in silico* analysis. QS drafted the manuscript. YJ reviewed and edited the manuscript. All authors contributed to the article and approved the submitted version.

## Funding

This work was supported by the National Natural Science Foundation of China (grant nos. 81830069 and 81861138054).

## Conflict of Interest

The authors declare that the research was conducted in the absence of any commercial or financial relationships that could be construed as a potential conflict of interest.

## Publisher’s Note

All claims expressed in this article are solely those of the authors and do not necessarily represent those of their affiliated organizations, or those of the publisher, the editors and the reviewers. Any product that may be evaluated in this article, or claim that may be made by its manufacturer, is not guaranteed or endorsed by the publisher.

## Footnotes

^1^ https://github.com/tseemann/shovill

^2^ http://www.genomicepidemiology.org/

^3^ https://pathogen.watch

^4^ https://github.com/tseemann/abricate

^5^ http://www-is.biotoul.fr

^6^ https://github.com/tseemann/snippy

^7^ https://github.com/tseemann/snp-dists

^8^ http://www.chinets.com/

## Supplementary Material

Supplementary Table S1 The primers used in this study.

Supplementary Figure S1 The resistance rates for ceftriaxone, chloramphenicol, ciprofloxacin, levofloxacin, ampicillin, and SMZ in different age of children, p<0.05 was labelled.

## References

[ref3] Carattoli A. Zankari E. Garcia-Fernandez A. Voldby Larsen M. Lund O. Villa L.. (2014). In silico detection and typing of plasmids using PlasmidFinder and plasmid multilocus sequence typing. Antimicrob. Agents Chemother. 58, 3895–3903. doi: 10.1128/AAC.02412-14, PMID: 24777092 PMC4068535

[ref4] Chang Y. J. Chen Y. C. Chen N. W. Hsu Y. J. Chu H. H. Chen C. L.. (2021). Changing antimicrobial resistance and epidemiology of non-Typhoidal Salmonella infection in Taiwanese children. Front. Microbiol. 12:648008. doi: 10.3389/fmicb.2021.648008, PMID: 33868207 PMC8044818

[ref6] Chen P. L. Li C. Y. Hsieh T. H. Chang C. M. Lee H. C. Lee N. Y.. (2012). Epidemiology, disease spectrum and economic burden of non-typhoidal Salmonella infections in Taiwan, 2006-2008. Epidemiol. Infect. 140, 2256–2263. doi: 10.1017/S0950268812000088, PMID: 22309742 PMC9152338

[ref7] EUCAST (2021). Breakpoint Tables for Interpretation of MICs and Zone Diameters, Version 11.0 [Online]. Available at: http://www.eucast.org/clinical_breakpoints/

[ref8] Feldgarden M. Brover V. Haft D. H. Prasad A. B. Slotta D. J. Tolstoy I.. (2019). Validating the AMRFinder tool and resistance gene database by using antimicrobial resistance genotype-phenotype correlations in a collection of isolates. Antimicrob. Agents Chemother. 63, e00483–e00419. doi: 10.1128/AAC.00483-19, PMID: 31427293 PMC6811410

[ref10] Haeusler G. M. Curtis N. (2013). Non-typhoidal Salmonella in children: microbiology, epidemiology and treatment. Adv. Exp. Med. Biol. 764, 13–26. doi: 10.1007/978-1-4614-4726-9_2, PMID: 23654054

[ref11] He D. D. Cui M. M. Zhang T. L. Hu G. Z. Liu J. H. Pan Y. S. (2021). Characterization of blaCMY-2-carrying IncC and rmtB-carrying IncI1/ST136 plasmids in an avian *Escherichia coli* ST224 strain. Plasmid 114:102555. doi: 10.1016/j.plasmid.2021.102555, PMID: 33472047

[ref13] Larsen M. V. Cosentino S. Rasmussen S. Friis C. Hasman H. Marvig R. L.. (2012). Multilocus sequence typing of total-genome-sequenced bacteria. J. Clin. Microbiol. 50, 1355–1361. doi: 10.1128/JCM.06094-11, PMID: 22238442 PMC3318499

[ref14] Li C. Zhang Z. Xu X. He S. Zhao X. Cui Y.. (2021). Molecular characterization of cephalosporin-resistant Salmonella Enteritidis ST11 isolates carrying blaCTX-M from children with diarrhea. Foodborne Pathog. Dis. 18, 702–711. doi: 10.1089/fpd.2020.2878, PMID: 33534635

[ref15] Liang Z. Ke B. Deng X. Liang J. Ran L. Lu L.. (2015). Serotypes, seasonal trends, and antibiotic resistance of non-typhoidal Salmonella from human patients in Guangdong Province, China, 2009-2012. BMC Infect. Dis. 15:53. doi: 10.1186/s12879-015-0784-4, PMID: 25881319 PMC4343067

[ref16] Lu X. Zeng M. Xu J. Zhou H. Gu B. Li Z.. (2019). Epidemiologic and genomic insights on mcr-1-harbouring Salmonella from diarrhoeal outpatients in Shanghai, China, 2006-2016. EBioMedicine 42, 133–144. doi: 10.1016/j.ebiom.2019.03.006, PMID: 30905850 PMC6491383

[ref17] Majowicz S. E. Musto J. Scallan E. Angulo F. J. Kirk M. O'brien S. J.. (2010). The global burden of nontyphoidal Salmonella gastroenteritis. Clin. Infect. Dis. 50, 882–889. doi: 10.1086/650733, PMID: 20158401

[ref18] Medalla F. Gu W. Mahon B. E. Judd M. Folster J. Griffin P. M.. (2016). Estimated incidence of antimicrobial drug-resistant nontyphoidal Salmonella infections, United States, 2004-2012. Emerg. Infect. Dis. 23, 29–37. doi: 10.3201/eid2301.160771, PMID: 27983506 PMC5176233

[ref19] Moon D. C. Kim S. J. Mechesso A. F. Kang H. Y. Song H. J. Choi J. H.. (2021). Mobile colistin resistance gene mcr-1 detected on an IncI2 plasmid in Salmonella Typhimurium sequence type 19 from a healthy pig in South Korea. Microorganisms 9:398. doi: 10.3390/microorganisms9020398, PMID: 33671955 PMC7919004

[ref20] Nahata M. C. Barson W. J. (1985). Ceftriaxone: a third-generation cephalosporin. Drug Intell. Clin. Pharm. 19, 900–906. doi: 10.1177/106002808501901203, PMID: 3910386

[ref21] Nguyen M. N. Hoang H. T. T. Xavier B. B. Lammens C. Le H. T. Hoang N. T. B.. (2021). Prospective one health genetic surveillance in Vietnam identifies distinct blaCTX-M-harbouring *Escherichia coli* in food-chain and human-derived samples. Clin. Microbiol. Infect. 27, 1515.e1–1515.e8. doi: 10.1016/j.cmi.2021.01.006, PMID: 33476808

[ref22] Paterson D. L. (2000). Recommendation for treatment of severe infections caused by Enterobacteriaceae producing extended-spectrum beta-lactamases (ESBLs). Clin. Microbiol. Infect. 6, 460–463. doi: 10.1046/j.1469-0691.2000.00107.x, PMID: 11168179

[ref23] Pragasam A. K. Anandan S. John J. Neeravi A. Narasimman V. Muthuirulandi Sethuvel D. P.. (2019). An emerging threat of ceftriaxone-resistant non-typhoidal Salmonella in South India: incidence and molecular profile. Indian J. Med. Microbiol. 37, 198–202. doi: 10.4103/ijmm.IJMM_19_300, PMID: 31745019

[ref24] Rau R. B. De Lima-Morales D. Wink P. L. Ribeiro A. R. Barth A. L. (2020). Salmonella enterica mcr-1 positive from food in Brazil: detection and characterization. Foodborne Pathog. Dis. 17, 202–208. doi: 10.1089/fpd.2019.2700, PMID: 31556704

[ref26] Rawat D. Nair D. (2010). Extended-spectrum beta-lactamases in Gram Negative bacteria. J. Glob. Infect. Dis. 2, 263–274. doi: 10.4103/0974-777X.68531, PMID: 20927289 PMC2946684

[ref27] Rodriguez-Urrego J. Herrera-Leon S. Echeita-Sarriondia A. Soler P. Simon F. Mateo S.. (2010). Nationwide outbreak of Salmonella serotype Kedougou associated with infant formula, Spain, 2008. Euro Surveill. 15:19582. doi: 10.2807/ese.15.22.19582-en, PMID: 20546688

[ref28] Seemann T. (2014). Prokka: rapid prokaryotic genome annotation. Bioinformatics 30, 2068–2069. doi: 10.1093/bioinformatics/btu153, PMID: 24642063

[ref29] Shane A. L. Mody R. K. Crump J. A. Tarr P. I. Steiner T. S. Kotloff K.. (2017). 2017 Infectious Diseases Society of America Clinical Practice Guidelines for the diagnosis and management of infectious diarrhea. Clin. Infect. Dis. 65, e45–e80. doi: 10.1093/cid/cix669, PMID: 29053792 PMC5850553

[ref30] Shi Q. Quan J. Lan P. Huang D. Zhou J. Jiang Y.. (2020). Prevalence and characteristics of pks gene cluster harbouring Klebsiella pneumoniae from bloodstream infection in China. Epidemiol. Infect. 148:e69. doi: 10.1017/S0950268820000655, PMID: 32160933 PMC7118716

[ref31] Siguier P. Perochon J. Lestrade L. Mahillon J. Chandler M. (2006). ISfinder: the reference centre for bacterial insertion sequences. Nucleic Acids Res. 34, D32–D36. doi: 10.1093/nar/gkj014, PMID: 16381877 PMC1347377

[ref32] Sjolund-Karlsson M. Howie R. Krueger A. Rickert R. Pecic G. Lupoli K.. (2011). CTX-M-producing non-Typhi Salmonella spp. isolated from humans, United States. Emerg. Infect. Dis. 17, 97–99. doi: 10.3201/eid1701.100511, PMID: 21192864 PMC3204627

[ref33] Smith S. I. Seriki A. Ajayi A. (2016). Typhoidal and non-typhoidal Salmonella infections in Africa. Eur. J. Clin. Microbiol. Infect. Dis. 35, 1913–1922. doi: 10.1007/s10096-016-2760-3, PMID: 27562406

[ref34] Stahlmann R. Lode H. M. (2013). Risks associated with the therapeutic use of fluoroquinolones. Expert Opin. Drug Saf. 12, 497–505. doi: 10.1517/14740338.2013.796362, PMID: 23651367

[ref35] Su L. H. Wu T. L. Chia J. H. Chu C. Kuo A. J. Chiu C. H. (2005). Increasing ceftriaxone resistance in Salmonella isolates from a university hospital in Taiwan. J. Antimicrob. Chemother. 55, 846–852. doi: 10.1093/jac/dki116, PMID: 15872047

[ref36] Subramanian B. Gao S. Lercher M. J. Hu S. Chen W. H. (2019). Evolview v3: a webserver for visualization, annotation, and management of phylogenetic trees. Nucleic Acids Res. 47, W270–W275. doi: 10.1093/nar/gkz357, PMID: 31114888 PMC6602473

[ref37] Switt A. I. Soyer Y. Warnick L. D. Wiedmann M. (2009). Emergence, distribution, and molecular and phenotypic characteristics of Salmonella enterica serotype 4,5,12:i. Foodborne Pathog. Dis. 6, 407–415. doi: 10.1089/fpd.2008.0213, PMID: 19292687 PMC3186709

[ref38] Trujillo-Soto T. Machuca J. Arca-Suarez J. Rodriguez-Iglesias M. Galan-Sanchez F. (2019). Co-occurrence of mcr-1 and qnrS1 on an IncHI2 plasmid in clinical isolates of Salmonella Typhimurium in Spain. Vector Borne Zoonotic Dis. 19, 662–665. doi: 10.1089/vbz.2018.2398, PMID: 31145042

[ref39] Wegener H. C. Hald T. Lo Fo Wong D. Madsen M. Korsgaard H. Bager F.. (2003). Salmonella control programs in Denmark. Emerg. Infect. Dis. 9, 774–780. doi: 10.3201/eid0907.030024, PMID: 12890316 PMC3023435

[ref40] Yoshida C. E. Kruczkiewicz P. Laing C. R. Lingohr E. J. Gannon V. P. Nash J. H.. (2016). The Salmonella *in silico* typing resource (SISTR): An open web-accessible tool for rapidly typing and subtyping draft Salmonella genome assemblies. PLoS One 11:e0147101. doi: 10.1371/journal.pone.0147101, PMID: 26800248 PMC4723315

