# Supplementary material for: Prevalence and Characteristics of Ceftriaxone-Resistant Salmonella in Children’s Hospital in Hangzhou, China
Source: Front Microbiol. 2021 Nov 22;12:764787. doi: 10.3389/fmicb.2021.764787 (PMC8645868; doi:10.3389/fmicb.2021.764787)
Supplement: Supplementary Table S1 — The primers used in this study. [file Table_1.DOCX]

| Gene | primers | Primer sequence (5’-3’) |
| --- | --- | --- |
| *bla*_CTX-M-1_ | CTX-M-1-F | CAGCGCTTTTGCCGTCTAAG |
|  | CTX-M-1-R | GGCCCATGGTTAAAAAATCACTGC |
| *bla*_CTX-M-9_ | CTX-M-9-F | GTTACAGCCCTTCGGCGATGATTC |
|  | CTX-M-9-R | GCGCATGGTGACAAAGAGAGTGCAA |
| *bla*_TEM_ | TEM-F | TRCGTGTCGCCCTTATTCCC |
|  | TEM-R | TGCTTAATCAGTGAGGCACC |
| *bla*_CMY-2_ | CMY-2-F | GCCGTTGCCGTTATCTAC |
|  | CMY-2-R | AATCTTTTTGTTCGTTCTGCG |
