# Supplementary figures and images for: Prevalence and Characteristics of Ceftriaxone-Resistant Salmonella in Children’s Hospital in Hangzhou, China
Source: Front Microbiol. 2021 Nov 22;12:764787. doi: 10.3389/fmicb.2021.764787 (PMC8645868; doi:10.3389/fmicb.2021.764787)

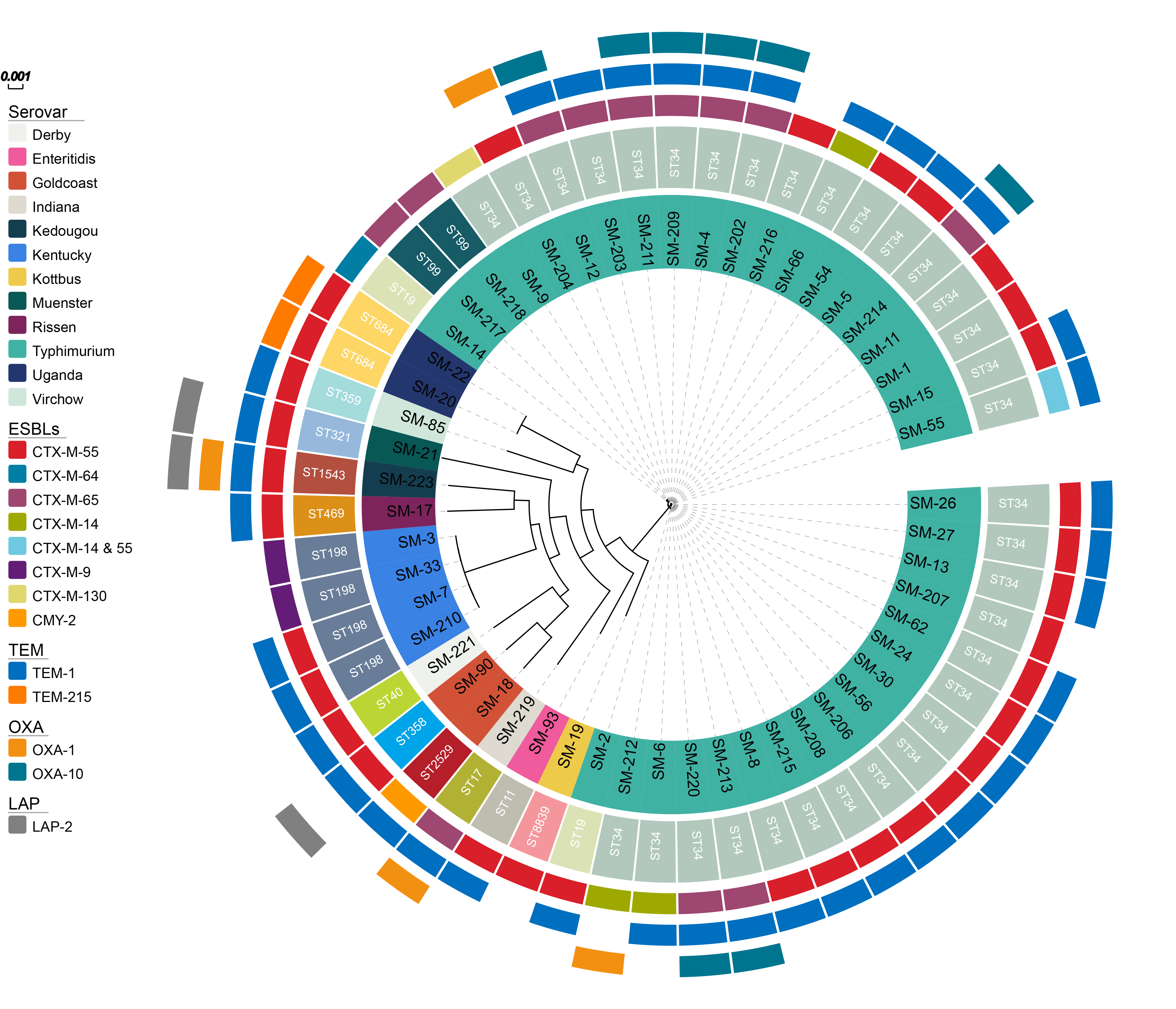

Supplement: Supplementary Figure S1 — The resistance rates for ceftriaxone, chloramphenicol, ciprofloxacin, levofloxacin, ampicillin, and SMZ in different age of children, p<0.05 was labelled. [file Image_1.TIF]
